# *Raveniolayangren* sp. n., a new troglobiontic spider from Hunan, China (Araneae, Nemesiidae)

**DOI:** 10.3897/BDJ.10.e85946

**Published:** 2022-05-30

**Authors:** Ye-Jie Lin, Xunyou Yan, Shuqiang Li

**Affiliations:** 1 Hebei Key Laboratory of Animal Diversity, College of Life Science, Langfang Normal University, Langfang 065000, China Hebei Key Laboratory of Animal Diversity, College of Life Science, Langfang Normal University Langfang 065000 China; 2 Institute of Zoology, Chinese Academy of sciences, Beijing, China Institute of Zoology, Chinese Academy of sciences Beijing China

**Keywords:** Asia, diagnosis, taxonomy, type

## Abstract

**Background:**

Troglomorphism is rare known in Mygalomorphae. Only three troglomorphic mygalomorphs have been recorded in China: *Raveniolabeelzebub* Lin & Li, 2020, *R.lamia* Yu & Zhang, 2021 and *Sinopesagollum* Lin & Li, 2021.

**New information:**

A new troglobiontic species of the genus *Raveniola* is described from China: *R.yangren* sp. n. (female) from Hunan. Photos and morphological description of the new species are given.This new species has elongated appendages and degenerated eyes in order to adapt to the cave environment. It can be distinguished by the stubby, unbranched spermathecae from other *Raveniola*.

## Introduction

Cave ecosystems have often evolved some special species. In Mygalomorphae, seven families have recorded troglomorphic species: *Troglodiplura* spp. (Anamidae); *Troglothelecoeca* Fage, 1929 (Barychelidae); *Harmoniconcerberus* Pedroso & Baptista, 2014, *Linothelecavicola* Goloboff, 1994, *Masteriacaeca* (Simon, 1892) and *M.pecki* Gertsch, 1982 (Dipluridae); *Euagrus* spp. (Euagridae); *Speloctenizaashmolei* Gertsch, 1982 (Microstigmatidae); *Raveniolabeelzebub* Lin & Li, 2020, *R.lamia* Yu & Zhang, 2021 and *Sinopesagollum* Lin & Li, 2021 (Nemesiidae); *Hemirrhagus* spp., *Holothelemaddeni* (Esposito & Agnarsson, 2014) and *Tmesiphanteshypogeus* Bertani, Bichuette & Pedroso, 2013 (Theraphosidae). Only three of them (*R.beelzebub*, *R.lamia* and *Sin.gollum*) have been recorded in China (Lin and Li 2020, Lin et al. 2021, Yu and Zhang 2021).

The spider family Nemesiidae Simon, 1889 currently includes 10 genera, 146 species and two fossil species of two genera from all continents, except Antarctica. Three genera occur in China: *Nemesia* Audouin, 1826 (*N.sinensis* Pocock, 1901 may belong to the Cyrtaucheniidae Simon, 1889 (Li and Zonstein 2015)), *Raveniola* Zonstein, 1987 and *Sinopesa* Raven & Schwendinger, 1995.

*Raveniola* (type species *Raveniolavirgata* (Simon, 1891)) is mainly distributed in China and Central Asia, included 42 species, with 21 species (50%) being described by both sexes and 16 species (38.1%) are endemic to China, most of them (13 species) being published after 2012. The species distribution of *Raveniola* in China is very uneven, eight species being distributed in Yunnan. This means that there will be a large number of new species to be discovered in other provinces (Li 2020, Yao et al. 2021, Li et al. 2021, Zhao et al. 2022, World Spider Catalog 2022). *Raveniola* can be distinguished from *Sinopesa* by the hirsute carapace, tarsal scopula well developed and male intercheliceral tumescence reduced or absent.

Here, we reported a new troglobiontic *Raveniola* species: *Raveniolayangren* sp. n. from Hunan, southeast China.

## Materials and methods

All specimens were preserved in 80% ethanol. The spermathecae were cleared in trypsin enzyme solution to dissolve non-chitinous tissues. Specimens were examined under a LEICA M205C stereomicroscope. Photomicrographs were taken with an Olympus C7070 zoom digital camera (7.1 megapixels). Laboratory habitus photographs were taken with a Sony A7RIV digital camera equipped with a Sony FE 90mm Goss lens. Photos were stacked with Helicon Focus (Version 7.6.1) or Zerene Stacker (Version 1.04) and processed in Adobe Photoshop CC2019.

All measurements are in millimetres and were obtained with an Olympus SZX16 stereomicroscope with a Zongyuan CCD industrial camera. Total length is measured without chelicerae. Trichobothria and spination were recorded for each segment, for example, Spination: leg III: Ti 1,2d; 1v; 1,2pl; Me 1,1(0)d; 1v; 1,1pl, which means on leg III, from terminal to basial, two rows of spines on dorsal tibia, first row with one spine, second row with two spines; one row of spine on ventral tibia, the row with one spine; two rows of spines on prolateral tibia, first row with one spine, second row with two spines; two rows of spines on dorsal metatarsus, both of them with one spine (on the other leg III, one row of spine on dorsal metatarsus, the row with one spine); one row of spines on ventral metatarsus, the row with one spine; two rows of spines on prolateral metatarsus, both of them with one spine. Trichobothria: leg I: Ta 1d(13), Me 1d(16), Ti 1pd-d(11), 1rd-d(11), which means on leg I, from terminal to basial, one row, with 13 trichobothria arranged on dorsal view of tarsus; one row, with 16 trichobothria arranged on dorsal view of metatarsus; two rows, one with 11 trichobothria arranged from prodorsal view to dorsal view, the other with 11 trichobothria arranged from retrodorsal view to dorsal view of tibia. Leg measurements are given as follows: total length (femur, patella, tibia, metatarsus, tarsus). Types of the new species reported here are deposited at the Institute of Zoology, Chinese Academy of Sciences in Beijing (IZCAS).

Abbreviations: **d** dorsal, **Fe** femur, **Me** metatarsus, **P** palp, **pd** prodorsal, **pl** prolateral, **rd** retrodorsal, **rl** retrolateral, **S** spermathecae, **Ta** tarsus, **Ti** tibia, **v** ventral.

## Taxon treatments

### Raveniola yangren

Lin & Li, 2022 sp. n.

12754A27-679C-5BF5-A6EF-382553CBE74D

53F39643-A624-4039-98E9-E884C812CC37

The nomenclature follows Zonstein and Marusik (2012).

#### Materials

- **Type status:** Holotype. **Occurrence:** recordedBy: Yejie Lin; individualID: IZCAS-Ar42725; individualCount: 1; sex: female; lifeStage: Adult; **Taxon:** kingdom:  Animalia; phylum: Arthropoda; class: Arachnida; order: Araneae; family: Nemesiidae; genus: Raveniola ; **Location:** continent: Asia; country: China; countryCode: CN; stateProvince: Hunan; county: Baojing; municipality: Xiangxi Tujia and Miao Autonomous Prefecture; locality: Wanmipo Town, a cave without name; verbatimElevation: 321 m; verbatimLatitude: 28.7749°N; verbatimLongitude: 109.4884°E; **Identification:** identifiedBy: Yejie Lin; **Event:** samplingProtocol: by hand; year: 2022; month: 4; day: 15; habitat: cave; eventRemarks: cave used as wine cellar, 16.5°C, RH 66.2%.
- **Type status:** Paratype. **Occurrence:** recordedBy: Yejie Lin; individualID: IZCAS-Ar42726, Ar42727; individualCount: 2; sex: females; lifeStage: Adults; **Taxon:** kingdom: Animalia; phylum: Arthropoda; class: Arachnida; order: Araneae; family: Nemesiidae; genus: Raveniola ; **Location:** continent: Asia; country: China; countryCode: CN; stateProvince: Hunan; county: Baojing; municipality: Xiangxi Tujia and Miao Autonomous Prefecture; locality: Wanmipo Town, a cave without name; verbatimElevation: 321 m; verbatimLatitude: 28.7749°N; verbatimLongitude: 109.4884°E; **Identification:** identifiedBy: Yejie Lin; **Event:** samplingProtocol: by hand; year: 2022; month: 4; day: 15; habitat: cave; eventRemarks: cave used as wine cellar, 16.5°C, RH 66.2%.
- **Type status:** Other material. **Occurrence:** recordedBy: Yejie Lin; individualCount: 6; sex: females; lifeStage: adults; **Taxon:** kingdom: Animalia; phylum: Arthropoda; class: Arachnida; order: Araneae; family: Nemesiidae; genus: Raveniola ; **Location:** continent: Asia; country: China; countryCode: CN; stateProvince: Hunan; county: Baojing; municipality: Xiangxi Tujia and Miao Autonomous Prefecture; locality: Wanmipo Town, a cave without name; verbatimElevation: 321 m; verbatimLatitude: 28.7749°N; verbatimLongitude: 109.4884°E; **Identification:** identifiedBy: Yejie Lin; **Event:** samplingProtocol: by hand; year: 2022; month: 4; day: 15; habitat: cave; eventRemarks: cave used as wine cellar, 16.5°C, RH 66.2%.
- **Type status:** Other material. **Occurrence:** recordedBy: Yejie Lin; individualCount: 23; lifeStage: juveniles; **Taxon:** kingdom: Animalia; phylum: Arthropoda; class: Arachnida; order: Araneae; family: Nemesiidae; genus: Raveniola ; **Location:** continent: Asia; country: China; countryCode: CN; stateProvince: Hunan; county: Baojing; municipality: Xiangxi Tujia and Miao Autonomous Prefecture; locality: Wanmipo Town, a cave without name; verbatimElevation: 321 m; verbatimLatitude: 28.7749°N; verbatimLongitude: 109.4884°E; **Identification:** identifiedBy: Yejie Lin; **Event:** samplingProtocol: by hand; year: 2022; month: 4; day: 15; habitat: cave; eventRemarks: cave used as wine cellar, 16.5°C, RH 66.2%.

#### Description

**Figure 1.**
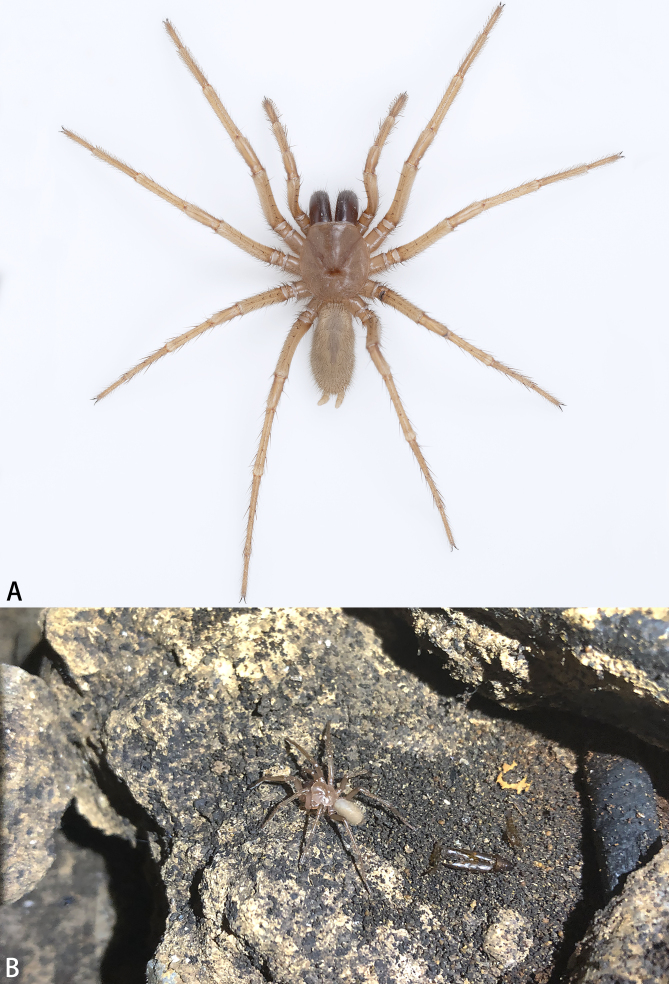
*Raveniolayangren* sp. n., live. **A** paratype, **B** holotype, in situ.

Female (Holotype) : Fig. 1.

**Figure 2.**
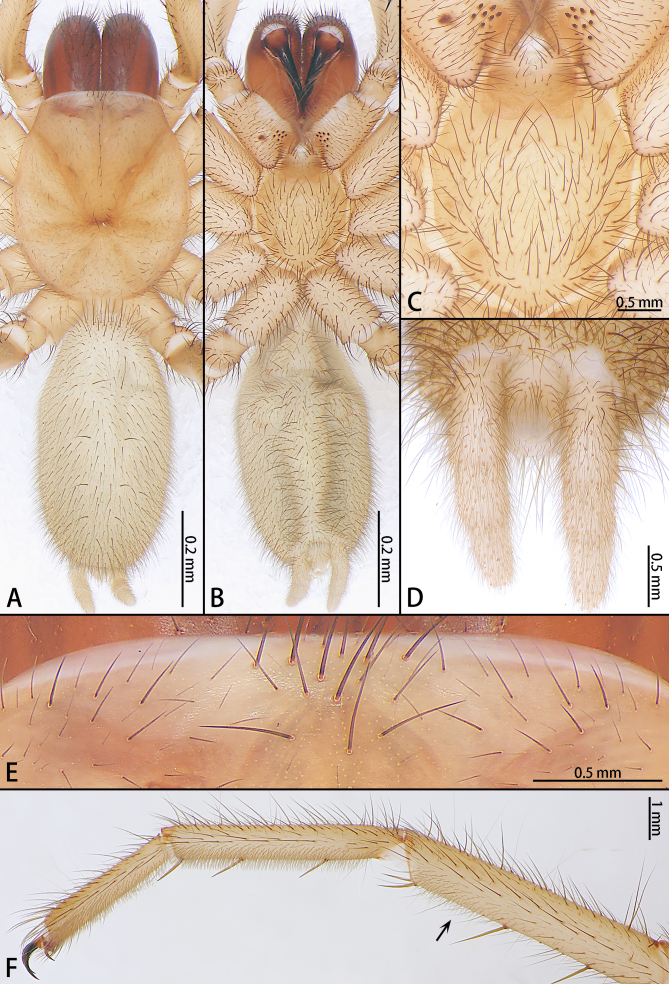
*Raveniolayangren* sp. n., holotype female. **A** habitus, dorsal, **B** same, ventral, **C** labium and sternum, **D** spinnerets, **E** ocular area, **F** leg I. Arrow shows scopula on tibia.

Colouration in alcohol: Carapace and legs pale yellow and abdomen grey. Chelicerae dark red (Fig. 2A, B).

Length: Total length 10.13.

Carapace: 4.43 long, 4.02 wide, 3.47 high, smooth, with two kinds of hairs: black erect bristles and fallen, slender, soft grey hairs; few bristles on margin. Eyes absent, the eye group area instead of eight bristles (Fig. 2E). The bristles in longitudinal row running from eye field to fovea. Fovea deep, straight, on the one third of carapace, occupying about one eighth of carapace width at that point. Four pairs of radial furrows, the colour of first pair of radial furrows darkest (Fig. 2A).

Chelicerae: 2.30 long, robust with long bristles dorsally and prolaterally. Promargin with dense brown hairs, retromargin with two rows of same sizes teeth: one row of ten teeth and the other with three denticles. Fang 1.89 long, basally dark brown, opening of the venom gland located dorsally.

Maxillae: 1.61 long, 0.94 wide, with 8/9 distinct black cuspules (Fig. 2C).

Labium: 0.87 long, 0.82 wide, basal with a pair of fan-shaped reticulation areas, terminal white, with long bristles (Fig. 2C).

Sternum: 2.50 long, 2.25 wide, covered with bristles of varying lengths, separated from labium by reticulation areas. Three pairs of sigilla, two anterior pairs small, oval; posterior pair of sigilla slightly larger than the previous two pairs (Fig. 2C).

Palp and legs: Tarsus and half part of tibia with scopula on palp; tarsus, metatarsus, and half part of tibia with scopula on leg I, II (Fig. 2F), scopula unobvious on leg III, IV. Trichobothria: Pa: Ta 1d(10), Ti 1d(8), 1d(8); leg I: Ta 1d(13), Me 1d(16), Ti 1pd-d(11), 1rd-d(11); leg II: Ta 1d(14), Me 1d(15), Ti 1pd-d(11), 1rd-d(11); leg III: Ta 1d(13), Me 1d(16), Ti 1pd-d(11), 1rd-d(11); leg IV: Ta 1d(16), Me 1d(15), Ti 1pd-d(11), 1rd-d(11). Spination: P: Ta 2v, Ti 4,2,3V, 1,1,1pl, Fe 2,1,1d; leg I: Me 2,2,1v, Ti 2,2,2v, Fe 1,1,1,0(1)d; leg II: Me 2,2,2v, Ti 2,2,2v, Fe 1,1,1,1d; leg III: Me 2,2,1d, 3,2,2v, 1,1rl, Ti 1,1d, 2,2,2v, 1,1rl, 1,1,1pl, Fe 2,2,2,1d; leg IV: Me 1d, 3,2,1(2),2v, 1,1,1(0)rl, 1(0)pl, Ti 1,1d, 3,2,2v, 1,1rl, 1,1,1pl, Fe 2,1,1,1d. Palp and legs measurements: P: 8.05 (2.78 + 1.07 + 2.30 + 1.90); leg I: 14.42 (4.18 + 1.98 + 3.68 + 2.70 + 1.88); leg II: 13.96 (3.86 + 2.04 + 3.29 + 2.79 + 1.98); leg III: 12.61 (3.11 + 1.33 + 3.09 + 3.24 + 1.84); leg IV: 16.41 (4.08 + 1.51 + 4.03 + 4.53 + 2.26). Leg formula: 4123.

Claw: Palp tarsus with one claw, leg tarsi with three claws, long and strongly curved. Proximal denticle on each side of paired tarsal claws. Denticle numbers: **P** 6; I 11, 11; II 11, 11; III 8, 9; IV 8, 7.

Abdomen: 5.29 long, 2.86 wide, oval, without any pattern, covered with bristles of varying lengths. Ten trichobothria in two rows longitudinal dorsally, basically distributed on both sides of the heart mark. Heart mark dark grey, sword-shaped (Fig. 2A).

Spinnerets: One pair of spinnerets (posterior lateral spinnerets), 2.20 long (proximal segment 0.77, median 0.51, distal 0.86), sclerotised weak (Fig. 2D).

**Figure 3.**
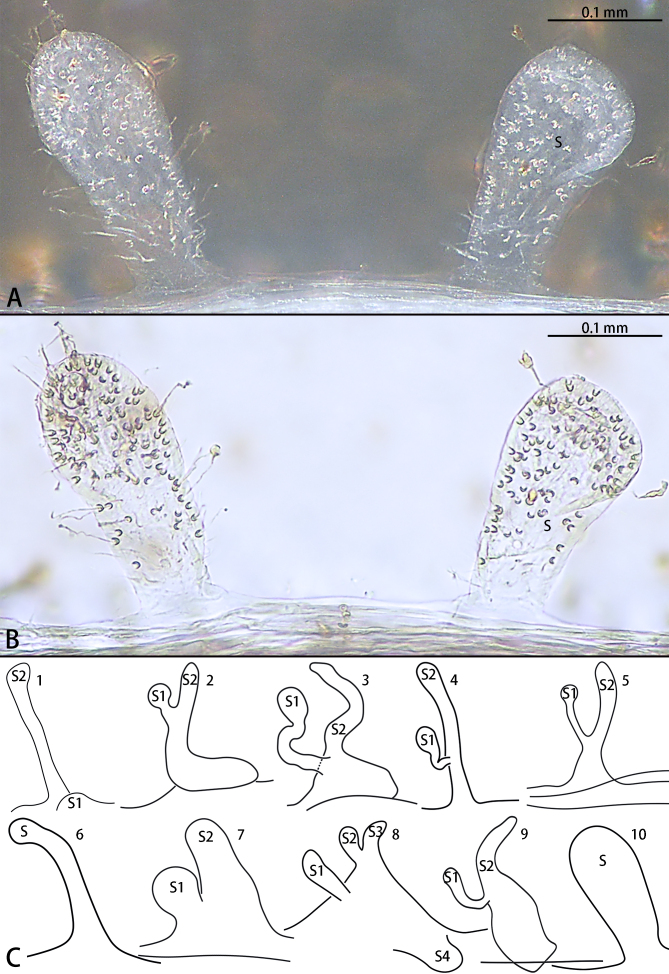
*Raveniola* spp., female genitalia, ventral view. **A**, **B** *R.yangren* sp. n., holotype female, **C-1** *R.beelzebub*, **C-2** *R.bellula* Li & Zonstein, 2015, **C-3** *R.chayi* Li & Zonstein, 2015, **C-4** *R.gracilis* Li & Zonstein, 2015, **C-5** *R.hebeinica* Zhu, F. Zhang & J. X. Zhang, 1999, **C-6** *R.lamia*, **C-7** *R.montana* Zonstein & Marusik, 2012, **C-8** *R.xizangensis* (Hu & Li, 1987), **C-9** *R.yajiangensis* Li & Zonstein, 2015, **C-10** *R.yangren* sp. n.

Vulva (Fig. 3A, B and C-10). Spermathecae white, two times longer than wide, head 1.5 times wider than the base of the stem; sclerotised weak. The pore glands more dense apically and becoming less numerous until the base. Apices broadly rounded. The ratio of the length of the spermathecae to the distance between the spermathecae is almost 1:1.

#### Diagnosis

The females of *Raveniolayangren* sp. n. resemble *R.beelzebub* Lin & Li, 2020 and *R.lamia* Yu & Zhang, 2021 by total reduction of eyes and the pale colouration (Fig. 2A, B and E). However, the new species can be distinguished by the spermathecae unbranched (vs. branched in *R.beelzebub*, other *Raveniola* spp. from China, see Fig. 3C) and the ratio of the length of spermathecae to the width is almost 2:1 (Fig. 3A and B) (vs. 8:1 in *R.lamia*, see Fig. 3C-6).

#### Etymology

The species is named after *Yangren*, a blind god who had hands with eyes in the palm in place of his normal eyes in Chinese traditional culture; noun in apposition.

#### Distribution

Known only from the type locality (China, Hunan).

#### Biology

Habitat under stones in the cave.

#### Taxon discussion

During the collection, 34 specimens of *Raveniolayangren* sp. nov were obtained, but no adult males were found. Therefore, the attribution cannot be determined from the male intercheliceral tumescence. We conclude from the hirsute carapace and well developed tarsal scopula that this new species should belong to *Raveniola*. At the same time, we note that the absence of PME in this new species is a distinctive feature. The presence of PMS was an important diagnostic feature in *Nemesia* versus *Iberesia*, but in *Raveniola*, this feature is highly variable. In *R.hebeinica*, the number of PME varies from 0 to 2 (Yu, personal communication). Therefore, we consider that the number of PME cannot be used as a character in *Raveniola*. For the above reasons, we classify this new species in *Raveniola* rather than independently as a new genus, although it has very specific cave-adapted characters.

## Supplementary Material

XML Treatment for Raveniola yangren
